# A Riboswitch-Based Inducible Gene Expression System for Mycobacteria

**DOI:** 10.1371/journal.pone.0029266

**Published:** 2012-01-18

**Authors:** Jessica C. Seeliger, Shana Topp, Kimberly M. Sogi, Mary L. Previti, Justin P. Gallivan, Carolyn R. Bertozzi

**Affiliations:** 1 Department of Pharmacological Sciences, Stony Brook University, Stony Brook, New York, United States of America; 2 Departments of Chemistry, University of California, Berkeley, California, United States of America; 3 Molecular and Cell Biology, University of California, Berkeley, California, United States of America; 4 Howard Hughes Medical Institute, University of California, Berkeley, California, United States of America; 5 Department of Chemistry and the Center for Fundamental and Applied Molecular Evolution, Emory University, Atlanta, Georgia, United States of America; Institut de Pharmacologie et de Biologie Structurale, France

## Abstract

Research on the human pathogen *Mycobacterium tuberculosis* (*Mtb*) would benefit from novel tools for regulated gene expression. Here we describe the characterization and application of a synthetic riboswitch-based system, which comprises a mycobacterial promoter for transcriptional control and a riboswitch for translational control. The system was used to induce and repress heterologous protein overexpression reversibly, to create a conditional gene knockdown, and to control gene expression in a macrophage infection model. Unlike existing systems for controlling gene expression in *Mtb*, the riboswitch does not require the co-expression of any accessory proteins: all of the regulatory machinery is encoded by a short DNA segment directly upstream of the target gene. The inducible riboswitch platform has the potential to be a powerful general strategy for creating customized gene regulation systems in *Mtb*.

## Introduction

Tools for manipulating gene expression are fundamental to genetic studies. Inducible systems, usually under the control of a small molecule, are particularly useful because they permit exquisite experimental control over both the dose and timing of gene expression. Inducible promoters are widely used to silence genes via direct transcriptional control or antisense methodologies and to overexpress proteins for biochemical and structural studies [1]. In bacteria, inducible systems have been used to elucidate gene function, determine gene essentiality, and validate drug targets [2], [3], [4].

Although several inducible expression systems exist for Gram-negative bacteria, adaptation to distantly related bacteria has proven difficult. Species that lack diverse regulated expression tools include the mycobacteria, among them *Mycobacterium tuberculosis* (*Mtb*), which causes tuberculosis in humans, and species that are commonly used as models for *Mtb* such as the fish and amphibian pathogen *M. marinum*, the non-pathogenic *M. smegmatis* (*Msmeg*), and the vaccine strain *M. bovis* BCG [5]. Unique challenges inherent to the biology of these medically relevant organisms, such as their pathogenesis, slow growth rate, and inefficient DNA uptake, have significantly hindered molecular genetics studies [6].

The earliest described mycobacterial inducible system, and the only one derived from endogenous mycobacterial machinery, is the acetamide-inducible *Msmeg* acetamidase promoter [7]. Although the system has proven useful for conditional mutant construction and protein overexpression, the acetamidase promoter exhibits a high level of basal activity and is prone to recombination in *Mtb* [7], [8], [9], [10]. Alternatives have been derived from transposons or the regulons of other Gram-positive bacteria [5], [11], [12], [13], [14], [15], including several tetracycline repressor-based (TetR) systems [16], [17], [18], [19].

For all of the above regulons, response to the inducer is mediated by one or more accessory proteins that must be imported into mycobacteria. Expression of these exogenous regulators can require optimization to achieve desired levels of induction [19]. Moreover, further adapting such systems can entail protein engineering and extensive characterization to verify function [20]. While these tools have proven useful in some contexts, alternative regulatory strategies, especially those that circumvent these limitations, would be valuable additions to the mycobacterial genetic toolkit.

**Figure 1:**
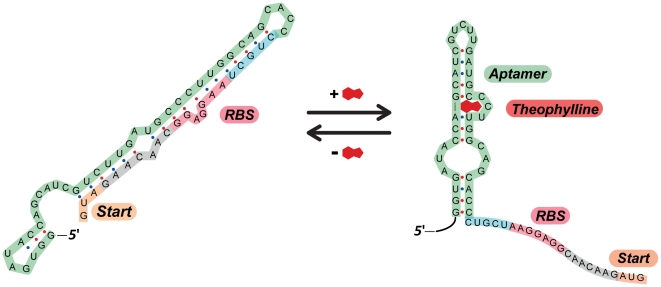
A theophylline-responsive riboswitch variant exerts translational control of gene expression. A synthetic theophylline-responsive riboswitch variant adopts a fold that sequesters the ribosome binding site (RBS) in the mRNA transcript. In the presence of theophylline, the riboswitch adopts a conformation in which the aptamer is bound to theophylline. The RBS is then released and able to promote protein translation. (The sequence for riboswitch E′ from ref [21] is depicted.)

We have recently reported a series of theophylline-responsive riboswitches that can control gene expression in a range of Gram-negative and Gram-positive bacteria, including *Msmeg*, a fast-growing, non-pathogenic species that is a widely used model system for *Mtb* [21]. Significantly, no exogenous regulator proteins are involved in the induction mechanism, making the system easy both to modify and to implement in different strains or species. The machinery is encoded by a single ∼300-bp DNA segment comprising a mycobacterial promoter (a variant of Phsp60, a widely used constitutive promoter from BCG [22]) and a synthetic RNA aptamer that binds to theophylline (Figure 1) [21]. Here we characterize this promoter-riboswitch combination and show that it can be used to induce and repress gene expression reversibly; to control a conditional gene knockdown; and to regulate expression in a macrophage model of infection.

## Results

### Characterization of riboswitch-controlled gene expression in mycobacteria

**Figure 2:**
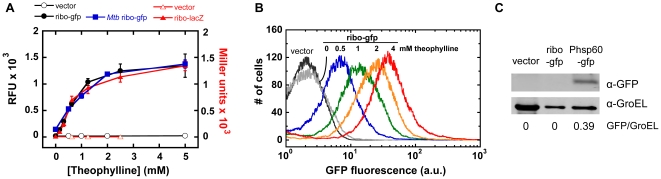
Theophylline induces riboswitch-mediated gene expression in *Msmeg* and *Mtb*. (A) Riboswitch-controlled GFP fluorescence in *Msmeg* (filled circles) and *Mtb* (filled squares) and β-galactosidase activity in *Msmeg* (filled triangles) in response to incubation in 0–5 mM theophylline for 6 h. Empty vector negative controls for GFP fluorescence and β-galactosidase activity are shown as open circles and triangles. Data are presented as relative fluorescence (RFU) for GFP and in Miller units for β-galactosidase, and as the mean ± SEM of three independent experiments. (B) Flow cytometry analysis of riboswitch-controlled GFP expression in *Msmeg* treated for 6 h with varying concentrations of theophylline. The empty vector control is shown in black. Results are representative of three or more independent experiments. (C) Immunoblot analysis of whole-cell lysates from *Mtb* harboring ribo-gfp, empty vector, or Phsp60-gfp positive control constructs. Band intensities were corrected for background, and GFP signal was normalized against the GroEL loading control.

To assess the generality of riboswitch response across different target genes, we created two constructs, ribo-gfp and ribo-lacZ, which are designed to express GFP and β-galactosidase under control of the riboswitch, and assayed their fluorescence or enzyme activity. With both constructs, we observed dose-dependent induction by theophylline with maximum reporter expression at ∼2 mM theophylline (Figure 2A), whereas significant growth attenuation was only observed at ≥10 mM theophylline in *Msmeg* (Figure S1). Also, the activation ratio, defined as the ratio of the reporter gene readout at 2 mM vs. 0 mM theophylline, was similar for the two reporter genes (89±12 for β-galactosidase and 65±8 for GFP). *Msmeg* ribo-gfp cells were further characterized by flow cytometry after incubation in 0–4 mM theophylline (Figure 2B). Each sample comprised a single distribution, and the mean GFP fluorescence intensity increased with theophylline concentration (Table S1).

In *Mtb* containing ribo-gfp, a similar dose response was observed as in *Msmeg* (Figure 2A). Growth rates of *Mtb* were slightly more sensitive to theophylline, with attenuation in *Mtb* observed at ≥5 mM (Figure S1). For the negative controls in the absence of theophylline, a higher fluorescence signal was observed from *Mtb* than *Msmeg*, perhaps due to higher scattering from *Mtb*. While this resulted in a lower calculated activation ratio in *Mtb* (8.2±0.84), the overall dose response and maximum fluorescence levels were similar between the two species (Figure 2A). To confirm that GFP expression in *Mtb* is fully repressed in the absence of theophylline, anti-GFP immunoblots were performed on whole-cell lysates (Figure 2C). In the absence of theophylline, no signal above background was detected in cells containing ribo-gfp or vector only.

**Figure 3:**
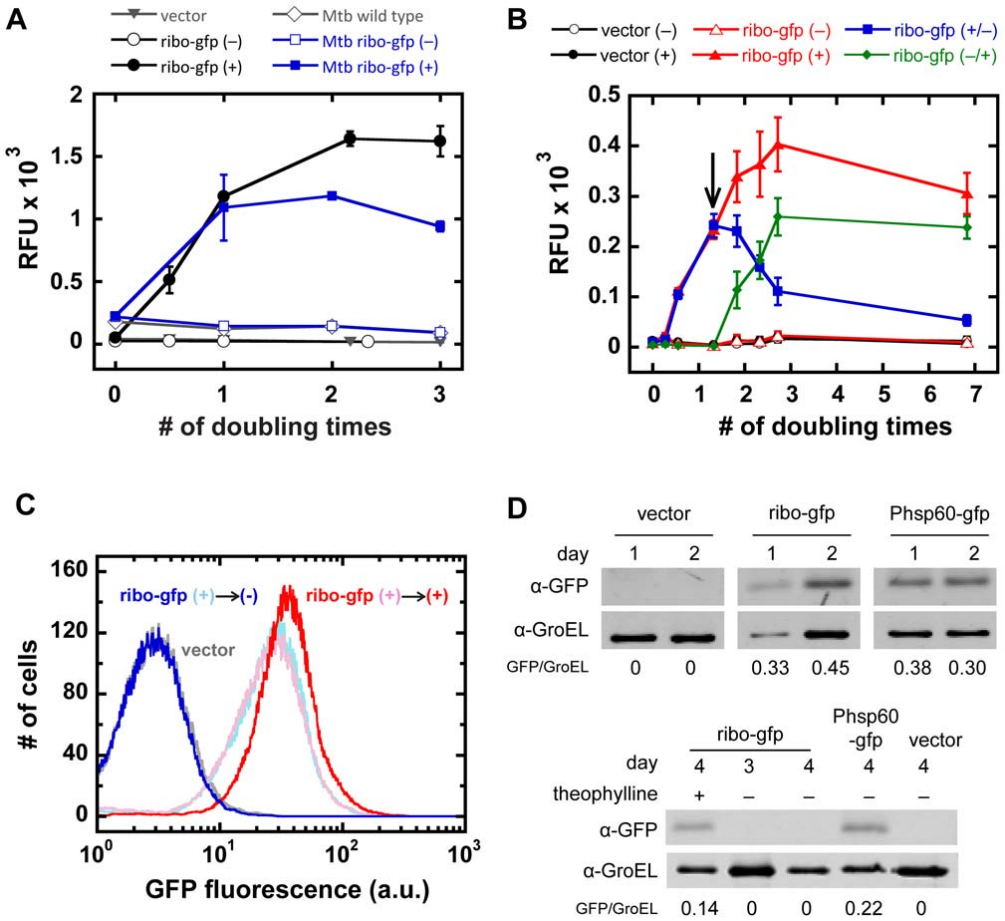
Theophylline riboswitch-controlled gene induction is reversible. (A) GFP fluorescence as a function of time in 0 mM (open) or 2 mM (filled) theophylline for *Msmeg* (circles) and *Mtb* (squares) harboring ribo-gfp. *Msmeg* vector and *Mtb* wild -type controls are shown as triangles and diamonds. Doubling times for *Msmeg* and *Mtb* are approximately 3 and 24 h, respectively. Data are presented as mean ± SEM of three independent experiments. GFP fluorescence from *Msmeg*::ribo-gfp and vector control strains was (B) monitored over time and (C) analyzed by flow cytometry after incubation with (+) or without (−) 2 mM theophylline. Theophylline was maintained or removed by media exchange after 1.3 doubling times (4 h; arrow). Kinetic data are presented as the mean ± SEM of eight replicates for each sample and are representative of three independent experiments. (D) Immunoblot analysis shows GFP induction in *Mtb* whole-cell lysates after incubation in 2 mM theophylline for one and two days (*top*). On day 2, theophylline was maintained (+) or removed by media exchange (−) and grown for an additional two days (*bottom*). Band intensities were corrected for background, and GFP signal was normalized against the GroEL loading control.

The kinetics of both gene expression and repression were measured in GFP fluorescence and immunoblot assays. In both *Mtb* and *Msmeg*, maximal GFP expression was observed after two doubling times (Figure 3A; as in Figure 2A, higher background fluorescence signal was detected for the *Mtb* vs. *Msmeg* negative controls). To demonstrate gene repression upon theophylline removal, *Msmeg* strains were induced for ∼1.3 doubling times (4 h) and then exchanged into fresh media with or without 2 mM theophylline. After theophylline removal, GFP fluorescence levels in *Msmeg* ribo-gfp cells were significantly reduced within five doubling times, whereas cells with theophylline maintained high GFP expression over the same period (Figure 3B; note that the data in Figures 3A and 2B were acquired on a different instruments, so RFU values cannot be directly compared between the two). These data, particularly the reversibility of theophylline induction, were further confirmed by flow cytometry. After the 4-h initial induction period, two identically induced ribo-gfp cell populations exhibited the same level of GFP expression. Five doubling times after the media exchange, ribo-gfp cells without theophylline were indistinguishable from vector control, whereas continuously induced cells maintained GFP expression (Figure 3C, Table S1). In *Mtb* ribo-gfp cells, the effect of theophylline removal was analyzed by immunoblot. Whereas as GFP expression was maintained over 4 days (∼4 doubling times) for cells incubated in theophylline, no GFP was detected one and two days after theophylline removal (Figure 3D).

### Theophylline-dependent knockdown of katG in Msmeg

**Figure 4:**
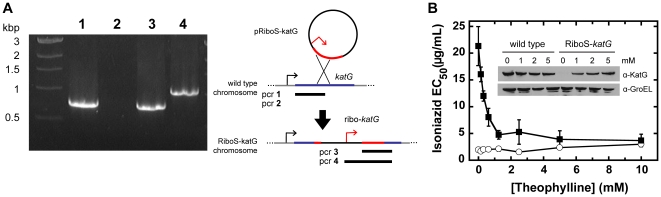
Theophylline controls endogenous KatG expression and restores sensitivity to isoniazid. (A) A single recombination event between the *Msmeg* chromosome and a plasmid containing the promoter-riboswitch combination and 500 bp of KatG yields the RiboS-*katG* strain containing a single full-length copy of *katG* under riboswitch control. The positive control for wild-type (**1**) and RiboS-katG (**3**) corresponds to the first 777 bp of *katG*. A primer specific to the promoter-riboswitch yields the predicted 1065-bp product from RiboS-*katG* (**4**), but not the wild type (**2**), confirming the recombination. (B) The isoniazid EC_50_ for *Msmeg* wild type (open circles) and RiboS-*katG* (filled squares) was measured in response to 0–10 mM theophylline. Data are presented as mean ± SEM of three independent experiments. (*inset*) The anti-KatG immunoblot for *Msmeg* wild type and RiboS-*katG* shows the response to 0–5 mM theophylline after 6 h. The GroEL immunoblot serves as a loading control, and data are representative of two independent experiments.

To assess the ability of the riboswitch-promoter combination to control theophylline-dependent knockdown mutants, we targeted *Msmeg katG* (MSMEG_6384), a homologue of *Mtb katG* (Rv1908c). The *katG* gene encodes a catalase-peroxidase that converts the prodrug isoniazid into its active form [23]. We generated a homologous recombinant strain, RiboS-*katG*, in which *katG* is under riboswitch regulation, and confirmed the single crossover event by PCR (Figure 4A).

For both the wild-type and RiboS-*katG* strains, the half-maximum effective concentration of isoniazid (EC_50_) was determined in a growth assay in 0–5 mM theophylline. RiboS-*katG* did not express KatG at levels detectable by immunoblot, indicating efficient repression in the absence of theophylline, and therefore exhibited isoniazid resistance compared to the wild type (EC_50_ ∼10 µg/mL) (Figure 4). In response to theophylline, dose-dependent KatG expression and increasing isoniazid sensitivity were observed. Addition of 2–5 mM theophylline induced KatG at levels sufficient to restore the wild-type isoniazid EC_50_.

### Theophylline-dependent expression in Mtb in a macrophage infection model

**Figure 5:**
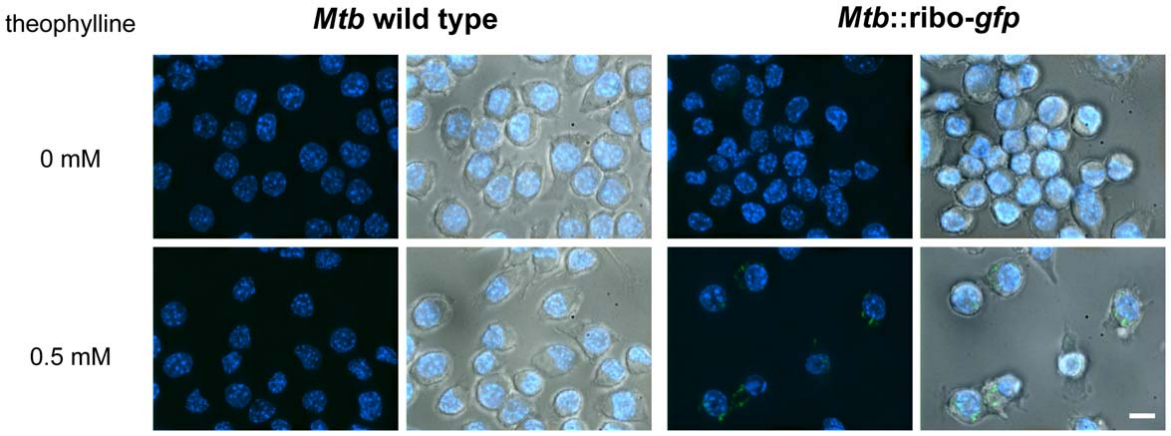
Theophylline induces riboswitch-controlled *Mtb* gene expression in a macrophage infection model. Murine macrophage-like RAW 264.7 cells infected with (A) *Mtb* wild type or (B) *Mtb*::ribo-gfp were induced with 0 mM or 0.5 mM theophylline for 24 h. Overlaid fluorescence signals from DAPI and GFP channels show nuclei (blue) and GFP-expressing bacteria (green). Panels on right show additional DIC light microscopy overlay. Scale bar represents 10 µm. Images are representative of three independent experiments for each condition.

To test the utility of the riboswitch regulatory system in the context of infection, we infected the murine macrophage-like RAW 264.7 cell line with *Mtb* harboring the ribo-gfp construct. Macrophages were then induced with 0 or 0.5 mM theophylline for one day, fixed, and imaged by fluorescence microscopy. GFP fluorescence was observed from intracellular *Mtb* containing ribo-gfp only in the presence of theophylline (Figure 5).

## Discussion

Both the activation ratio and time response of the riboswitch-based system compare favorably with the ∼100-fold activation ratios and 2-day induction times reported for the nitrile-inducible and *Tn10*-derived Tet systems [14], [19]. In a direct comparison between the riboswitch and Tet systems in *Msmeg*, the activation ratio for GFP was equivalent (69±3 for the riboswitch vs. 72±5 for Tet; data not shown). We further determined that the riboswitch functions as a titratable system, like the *Tn10* TetR repressor, rather than as a bistable switch, like the nitrile-inducible system [14]. Also, the effect of theophylline is reversible in both *Msmeg* and *Mtb* upon removal of the inducer.

Gene expression in an intracellular pathogen such as *Mtb* is often regulated in response to changes in the host environment, such as internalization by macrophages [24]. The ability to modulate expression levels during infection is critical to determining how specific genes affect bacterial survival and disease progression in the host. Chromosomal gene knockouts are commonly used to examine gene function or determine gene essentiality, and conditional gene knockdowns afford the additional power of inducing expression or repression at a defined phase of growth or infection. The results from the conditional *katG* knockdown and macrophage infection experiments show that the riboswitch affords control of mycobacterial gene expression both *in vitro* and within host macrophages.

We have confirmed that the riboswitch can regulate gene expression in both the model organism *Msmeg* and the pathogen *Mtb*, suggesting that the mechanism of riboswitch induction is species-independent and that factors affecting intracellular theophylline concentration—such as membrane penetration, metabolism, and efflux—operate similarly in both species. These data also demonstrate the consistency of riboswitch response to theophylline across a variety of *in vitro* and cell-based applications that are relevant to the study of *Mtb* and other mycobacteria. Finally, the similarity in responses shows that *Msmeg* can serve as a host for screening further iterations of riboswitch-based mycobacterial gene regulation. We anticipate that the construction of promoter-riboswitch libraries and the ability to screen by fluorescence, as demonstrated here, will facilitate the engineering of enhancements such as increased dynamic range, decreased basal expression, and inducible repression. Such screens could be performed not only under standard culture conditions, but also in a macrophage infection model or various *in vitro* culture models, such as hypoxia [25] and carbon starvation [26], that mimic tuberculosis disease states.

Application of the theophylline-responsive riboswitch system to animal models of infection would be facilitated by the fact that theophylline is an FDA-approved drug and well tolerated in mice and guinea pigs. However, because theophylline is a bronchodilator, its use may complicate *Mtb* infection studies. Given that the TetR system is the only mycobacterial inducible system currently available for use in animals, the application of theophylline riboswitch in animal models of tuberculosis nevertheless warrants further investigation.

## Materials and Methods

### Reporter gene assays for riboswitch-regulated constructs

See Tables S1, S2, and S3 for summaries of all strains, constructs and primers as well as Text S1 for detailed methods used in this study. Riboswitch-reporter plasmids were constructed by assembly PCR methods as previously described [21]. Whole-cell GFP fluorescence assays were performed as reported for *Msmeg* [21]. Briefly, for dose response curves, cultures were grown from early to late log phase (optical density at 600 nm [OD_600_] of 0.2 or 0.3 to ∼1) over two doubling times (6 h) in media containing 0–5 mM theophylline. Emission from whole-cell suspensions was measured at 510 nm with excitation at 450 nm and a 495 nm high-pass cutoff filter in a Gemini XPS fluorescence microplate reader (Molecular Devices Corporation). For *Mtb*, cells were resuspended and incubated at room temperature for 1 h in 200 µL phosphate-buffered 10% formalin prior to fluorescence measurement.

To measure the kinetics of GFP repression upon theophylline removal, the OD_600_ and GFP fluorescence of *Msmeg* cultures were monitored continuously in a 96-well plate. Briefly, bacteria were inoculated at OD_600_ of 0.1 in 300 µL in black, clear-bottom 96-well plates in media containing 0 or 2 mM theophylline and incubated without shaking at 37°C. After 4 h, cells were pelleted and exchanged into fresh media with or without theophylline and monitored for an additional 15–17 h. For these assays, fluorescence was measured with a FLUOstar Optima plate reader (BMG Labtech) with 485-nm excitation and 520-nm emission filters (30 flashes per well, constant gain of 1000). All GFP data are reported as relative fluorescence (RFU) normalized by the OD_600_ for each sample. β-Galactosidase activity in whole-cell lysates was measured as previously described [27].

### Flow cytometry

For each sample, 1–3×10^8^ *Msmeg* cells (based on OD_600_ of 1 = 3×10^8^ cells/mL) were washed twice with 1 mL of PBS and resuspended in 1 mL 10% formalin. After sonication in an ice water bath for 2 minutes, cell clumps were pelleted by centrifugation for 10 min at 200× g, and 900 µL supernatant was removed for analysis with a Becton Dickinson FACScan flow cytometer (Clinical Flow Cytometry Laboratory, Stony Brook University). For each experiment, the vector control sample was used to set a gate based on forward and side scatter channels to select against debris and any remaining cell clumps. Histograms were calculated from approximately 2×10^4^ cells per sample using Cyflogic software (CyFlo Ltd., Finland).

### Immunoblot analysis of *Mtb* lysates

*Mtb* strains were inoculated at OD_600_ of 0.05 in 30-mL cultures and incubated with shaking at 37°C in medium containing 2 mM theophylline. On day two, a subset of cultures was pelleted and resuspended in medium without theophylline, and all cultures were grown for an additional two days. On each day of the experiment, samples were removed for OD_600_ measurements and to obtain lysates for immunoblot analysis. Cells were killed by boiling and lysed by bead-beating (2×5k rpm for 30 s each). After removing cell debris by centrifugation, supernatants were stored at −80°C until further analysis.

Immunoblots were performed using the Odyssey Western Blotting kit III LT (LI-COR Biosciences). Where necessary, lysates were concentrated by centrifugal filtration. Two micrograms of protein (or, where the protein concentration was insufficient, the maximum volume per well) were separated by SDS-PAGE and transferred to nitrocellulose. Proteins were detected with anti-GFP (Invitrogen #33-2600 at 1∶1000 dilution for Figure 3D (*top*); Abcam #ab290 at 1∶100 dilution for Figure 3D (*bottom*)) and anti-GroEL2 (Abcam #ab20519 at 1∶200 dilution) antibodies. Membranes were probed with anti-mouse IgG-800CW (1∶15,000 dilution, #926-32212, LI-COR Biosciences) or anti-rabbit IgG-680LT (1∶20,000 dilution, #827-11081, LI-COR Biosciences) and imaged by infrared fluorescence detection. Quantitative analysis was performed using the Odyssey Imaging System software.

### Construction and characterization of Msmeg with katG under riboswitch control

A homologous recombinant *Msmeg* strain was generated as described using pRiboS-*katG*, a suicide vector containing a segment of the *Msmeg katG* gene MSMEG_6384 [28]. Homologous recombination at the *katG* locus was verified by PCR on genomic DNA isolated from a single clone. The half-maximum effective concentration of isoniazid (EC_50_) was determined in a growth assay at each theophylline concentration. Expression of KatG in *Msmeg* wild-type and RiboS-*katG* strains was confirmed in whole-cell lysates by immunoblot and chemiluminescent detection using anti-KatG (TB Vaccine Testing and Research Materials Contract HHSN266200400091c, Colorado State University) and anti-GroEL2 antibodies.

### Macrophage infection and microscopy

RAW 264.7 cells (ATCC TIB-71) were incubated in an *Mtb* suspension at an MOI of 5 for 4 h and washed with PBS. Infected macrophages were allowed to recover in medium until replacement at 24 h post infection with fresh medium containing 0 or 0.5 mM theophylline. After an additional day, macrophages were washed with PBS and fixed in phosphate-buffered 10% formalin for 1 h. Coverslips were mounted on glass slides with VectaShield mounting medium plus DAPI stain (Vector Laboratories). Image stacks were acquired on a Zeiss Axiovert 200 M using a 100×1.3 numerical aperture lens. Following digital deconvolution using the nearest-neighbors algorithm in Slidebook (Intelligent Imaging Solutions), final images were generated by z-projection of 30–40 frames at 0.34 µm separation.

## Supporting Information

Figure S1 **Growth of mycobacteria in theophylline.** (A) Growth in medium containing 0–20 mM theophylline was monitored for (A) *Mtb* and (B) *Msmeg*.
(TIF)

Text S1 **Detailed protocols for DNA constructs and assays.**
(DOC)

Table S1 **Mean fluorescence intensities for flow cytometry analysis of** ***Msmeg*** **.**
(DOC)

Table S2 **Bacterial strains used in this study.**
(DOC)

Table S3 **Oligonucleotides used for PCR amplification or site-directed mutagenesis.**
(DOC)
