# Supplementary figures and images for: A Riboswitch-Based Inducible Gene Expression System for Mycobacteria
Source: PLoS One. 2012 Jan 18;7(1):e29266. doi: 10.1371/journal.pone.0029266 (PMC3261144; doi:10.1371/journal.pone.0029266)

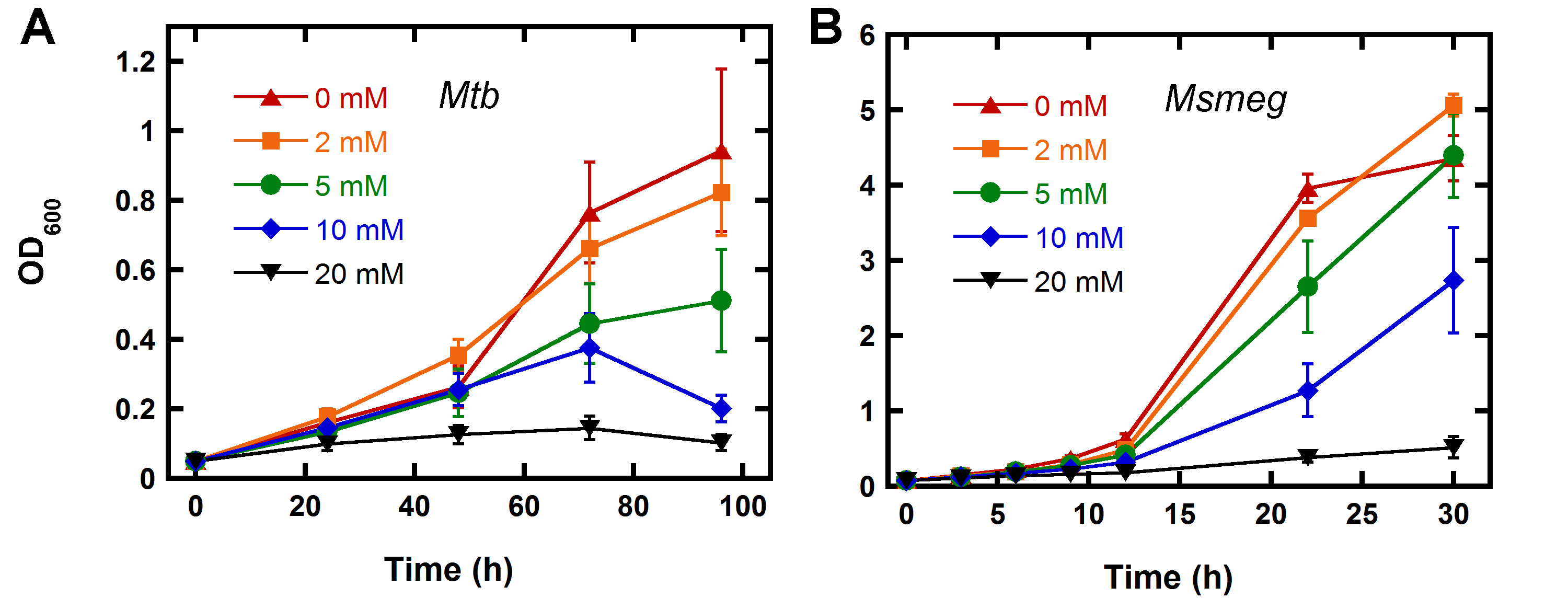

Supplement: Figure S1 — Growth of mycobacteria in theophylline. (A) Growth in medium containing 0–20 mM theophylline was monitored for (A) Mtb and (B) Msmeg. (TIF) [file pone.0029266.s001.tif]
