# Supplementary material for: A Riboswitch-Based Inducible Gene Expression System for Mycobacteria
Source: PLoS One. 2012 Jan 18;7(1):e29266. doi: 10.1371/journal.pone.0029266 (PMC3261144; doi:10.1371/journal.pone.0029266)
Supplement: Text S1 — Detailed protocols for DNA constructs and assays. (DOC) [file pone.0029266.s002.doc]

**Text S1.** **Detailed protocols for DNA constructs and assays.**

### Growth conditions

*Msmeg* mc^2^155 and *Mtb* H37Rv strains were used as wild type and for all experiments below. The growth medium was 7H9 (liquid) or 7H11 (solid) with 0.5% glycerol and 0.05% Tween-80 supplemented with 10% ADC (*Msmeg*) or 10% OADC (*Mtb*) supplemented with 20 μg/mL kanamycin. The RAW 264.7 murine macrophage-like cell line (ATCC #TIB-71) was cultured in RAW media (RPMI-1640 plus L-glutamine and 10% fetal bovine serum) unless otherwise noted.

### Contruction of plasmids used in this study

The *egfp* gene (hereafter referred to as gfp) encoding the fluorescence-enhancing mutations F64L and S65T was subcloned from pEGFP-N1 (Clontech) into the mycobacterial shuttle plasmid pMV261 to create pMWS114. The construction of pST5552 (ribo-gfp) based on pMWS114 was previously described [1]. Briefly, the 5’ untranslated region (168 bp upstream of the start codon) is predicted by the mFold program to be highly structured and could interfere with riboswitch function[2]. This portion of the *M. bovis* BCG hsp60 promoter (Phsp60) was removed and replaced by assembly PCR methods with a theophylline riboswitch. The ribo-lacZ construct was obtained by similar methods based on a vector with the lacZ gene cloned from pSKD345.1 into pMWS114.

pRibo was created by PCR-based site-directed mutagenesis of pST5552 to delete the gfp gene and simultaneously insert a BsaI restriction site immediately following the start codon to generate pRibo. The mycobacterial origin of replication (oriM) was removed in a second mutagenesis step to create the plasmid pRiboS, which cannot replicate in mycobacteria. The first 720 bp of katG (MSMEG_6384; GeneID 4536370) plus the stop codon TAA were PCR-amplified from *Msmeg* genomic DNA[4] and ligated into pRiboS using BsaI to create pRiboS-katG.

### Theophylline response assays

*Msmeg* transformed with pMV261 (vector) or pST5832 (ribo-lacZ) was grown and harvested as for the GFP assay. Cell pellets were resuspended in 1 mL Z buffer (60 mM Na_2_HPO_4_, 40 mM NaH_2_PO_4_, 10 mM KCl, 1 mM MgSO_4_, 50 mM β-mercaptoethanol, pH 7.0). Cells were lysed with two pulses of 20 s each at power level 0.5 with a tip sonicator (Sonicator 3000, Misonix, Inc.). At t = 0 min, 50 μL 2-nitrophenyl β-D-galactopyranoside (4 mg/mL in Z buffer) was added to 200 μL cell lysate. Reactions were incubated at 30° C until yellow color was visible (~10 min.). After recording the time and stopping the reaction with 125 μL 1 M sodium bicarbonate, cell debris was pelleted and the final OD_420_ was recorded. Substrate turnover is reported in Miller units: (OD_420_ x 1000) / (OD_600_ x rxn time in min).

*Msmeg* wild-type and RiboS*-katG* strains were grown in 0-100 μg/mL isoniazid and 0-10 mM theophylline in 96-well plates without shaking, and the final OD_600_ recorded after 24 h. For each theophylline concentration, the OD_600_ as a function of isoniazid concentration was fit to a single exponential using Kaleidagraph (Synergy Software) to obtain the half-maximum effective concentration of isoniazid (EC_50_) at each theophylline concentration.

For immunoblots, *Msmeg* wild-type and RiboS*-katG* strains were grown to late-log phase and diluted to OD_600_ of 0.15 in 10 mL 7H9 with 0, 1, 2, or 5 mM theophylline. After 8 h incubation, cells were pelleted, resuspended in PBS + 5 μg/mL DNase, and lysed by microtip sonication (10 s on, 10 s off, 2 min total processing time). Cleared whole-cell lysate (30 μg per sample) was separated by SDS-PAGE (10% Criterion gel, Bio-Rad Laboratories), transferred, and probed with antibodies as described in the main text.

For *Mtb* infections, RAW 264.7 cells were seeded on 22 x 22 mm sterile glass coverslips in 6-well plates at 3 x 10^5^ cells per well and grown for 1 day. *Mtb* wild type and *Mtb*::ribo-gfp were grown to late log phase. An aliquot of each culture was spun at 500 x g for 5 min to remove cell clumps, and the supernatant was transferred to a fresh tube and spun at 3500 x g for 5 min. The resulting cell pellet was washed twice with equal volumes of PBS and then diluted in the appropriate volume of RAW medium (10% horse serum instead of fetal calf serum) for a multiplicity of infection (MOI) of 5 bacteria per macrophage.

## References

1. Topp S, Reynoso CK, Seeliger JC, Goldlust IS, Desai SK, et al. (2010) Synthetic Riboswitches that Induce Gene Expression in Diverse Bacterial Species. Applied and Environmental Microbiology 76: 7881-7884.

2. Zuker M (2003) Mfold web server for nucleic acid folding and hybridization prediction. Nucl Acids Res 31: 3406-3415.

3. Ehrt S, Guo XV, Hickey CM, Ryou M, Monteleone M, et al. (2005) Controlling gene expression in mycobacteria with anhydrotetracycline and Tet repressor. Nucl Acids Res 33: e21-.

4. Larsen M, Biermann KE, Tandberg S, Hsu T, Jacobs Jr WR (2007) Genetic Manipulation of *Mycbacterium tuberculosis*. Current Protocols in Microbiology 10A: 2.1-2.21.
