# Supplementary material for: A Riboswitch-Based Inducible Gene Expression System for Mycobacteria
Source: PLoS One. 2012 Jan 18;7(1):e29266. doi: 10.1371/journal.pone.0029266 (PMC3261144; doi:10.1371/journal.pone.0029266)
Supplement: Table S1 — Mean fluorescence intensities for flow cytometry analysis of Msmeg . (DOC) [file pone.0029266.s003.doc]

**Table S1. Mean fluorescence intensities from flow cytometry analysis of *Msmeg.***

| **Figure** | **Sample** | **[Theophylline]** | **MFI** |
| --- | --- | --- | --- |
| **1B** | vector |  | 2.12 |
|  | ribo-gfp | 0 mM | 2.09 |
|  | ribo-gfp | 0.5 mM | 5.85 |
|  | ribo-gfp | 1 mM | 11.89 |
|  | ribo-gfp | 2 mM | 20.05 |
|  | ribo-gfp | 4 mM | 26.52 |
|  |  |  |  |
| **2C** | vector |  | 3.07 |
|  | ribo-gfp | **2** 🡪 0 mM | 24.74 |
|  |  | 2 🡪 **0 mM** | 2.95 |
|  | ribo-gfp | **2** 🡪 2 mM | 23.36 |
|  |  | 2 🡪 **2 mM** | 35.86 |
