# Supplementary material for: A Riboswitch-Based Inducible Gene Expression System for Mycobacteria
Source: PLoS One. 2012 Jan 18;7(1):e29266. doi: 10.1371/journal.pone.0029266 (PMC3261144; doi:10.1371/journal.pone.0029266)
Supplement: Table S2 — Bacterial strains used in this study. (DOC) [file pone.0029266.s004.doc]

**Table S2. Bacterial strains and plasmids used in this study**

| **Strains** |  | **Genotype** | **Source** |
| --- | --- | --- | --- |
| *E. coli* |  |  |  |
| *M. tuberculosis* H37Rv |  |  |  |
| *M. smegmatis* mc^2^155 |  |  |  |
| *M. smegmatis* mc^2^155 | RiboS-*katG* | Kn^r^, contains one full-length copy of katG under riboswitch control | This work |
| **Plasmids** |  |  |  |
| **Reference name** | **Name** | **Description** | **Source** |
|  | pMV261 | Kn^r^, pAL5000 origin, ColE1 origin, multiple cloning site, Phsp60 promoter | Ref [1] |
|  | pMWS114 | pMV261 derivative, contains *egfp* | This work |
|  | pSKD345.1 | pLacZU1hpII derivative; contains *lacZ* gene | Ref [2] |
|  |  |  |  |
| ribo-gfp | pST5552 | pMWS114 derivative; Phsp60 -riboE' hybrid, *egfp* | Ref [3] |
| ribo-lacZ | pST5832 | pST5552 derivative; Phsp60 -riboE' hybrid, *lacZ* | This work |
|  | pRibo | pST5552 derivative; lacks *gfp* gene, includes BsaI site for cloning | This work |
|  | pRiboS | pRibo derivative; lacks pAL5000 origin, cannot replicate in mycobacteria | This work |
|  | pRiboS-*katG* | pRiboS derivative; contains first 720bp of *Msmeg* *katG* (MSMEG_6384) | This work |

**References**

1. Stover CK, de la Cruz VF, Fuerst TR, Burlein JE, Benson LA, et al. (1991) New use of BCG for recombinant vaccines. Nature 351: 456-460.

2. Desai SK, Gallivan JP (2004) Genetic screens and selections for small molecules based on a synthetic riboswitch that activates protein translation. J Am Chem Soc 126: 13247-13254.

3. Topp S, Reynoso CK, Seeliger JC, Goldlust IS, Desai SK, et al. (2010) Synthetic Riboswitches that Induce Gene Expression in Diverse Bacterial Species. Appl Environ Microbiol 76: 7881-7884.
