# Supplementary material for: A Riboswitch-Based Inducible Gene Expression System for Mycobacteria
Source: PLoS One. 2012 Jan 18;7(1):e29266. doi: 10.1371/journal.pone.0029266 (PMC3261144; doi:10.1371/journal.pone.0029266)
Supplement: Table S3 — Oligonucleotides used for PCR amplification or site-directed mutagenesis. (DOC) [file pone.0029266.s005.doc]

**Table S3. Oligonucleotides used for PCR amplification or site-directed mutagenesis.**

Unique restriction sites are underlined.

| **Target** | **Template** | **Product** | **Forward Primer (5' -> 3')** | **Reverse Primer (5' -> 3')** |
| --- | --- | --- | --- | --- |
| *egfp* | pEGFP-N1 (Clontech) | pMWS114 | CCAGCTGCAGAATTCGTGAGCAAGGGCGAGGAGCT | TGGACGAGCTGTACAAGTAAAAGCTTATCGATGTCG |
| *lacZ* | pSKD345.1 | pHsp-*lacZ* | CGCATGGCCAGCACCATGATTACGGATTCACTGGC | GGGAAGCTTcTATTTTTGACACCAGACCAACTGG |
| delete *egfp*,  add BsaI site | pST5552 | pRibo | CAACAAGATGGAGACCAAGACAATTGCGGATCCAGC | GCAATTGTCTTGGTCTCCATCTTGTTGCCTCCTTAGC |
| delete *oriM* | pRibo | pRiboS | GAGCGCAACGCGTGCGGCCGCGGTACCAGATC | GCGGCCGCACGCGTTGCGCTCGGTCGTTCG |
| *katG* 5' 720 bp | *M. smegmatis* genomic DNA | pRiboS-*katG* | AAAGGTCTCAGATGCCTGAGGATCGCCCGATCG | TTTGGTCTCTAGCTTAGCCTTCGGGGTTGACG |
|  |  |  |  |  |
| **For PCR verification of RiboS-*katG* strain (see Figure 4A)** | | | |  |
| *katG* |  | pcr 1, pcr 3 | GCACGAACTTCCCGATCACAC | GAACGTCTCGCGGATGTCGA |
| RiboS-*katG* |  | pcr 2, pcr 4 | CTAGAGGTGACCACAACG | GAACGTCTCGCGGATGTCGA |
